# Eliminate mitochondrial diseases by gene editing in germ-line cells and embryos

**DOI:** 10.1007/s13238-015-0177-x

**Published:** 2015-06-17

**Authors:** Si Wang, Fei Yi, Jing Qu

**Affiliations:** National Laboratory of Biomacromolecules, Institute of Biophysics, Chinese Academy of Sciences, Beijing, 100101 China; Department of Molecular and Cellular Physiology, Stanford University School of Medicine, Stanford, CA 94305 USA; State Key Laboratory of Reproductive Biology, Institute of Zoology, Chinese Academy of Sciences, Beijing, 100101 China

## Abstract

Nuclease-based gene editing technologies have opened up opportunities for correcting human genetic diseases. For the first time, scientists achieved targeted gene editing of mitochondrial DNA in mouse oocytes fused with patient cells. This fascinating progression may encourage the development of novel therapy for human maternally inherent mitochondrial diseases.

In recent years, remarkable advances in nuclease-based genome editing technologies including helper-dependent adenovirus vector (HDAdV), zinc finger nuclease (ZFN), transcriptional activator-like effector nucleases (TALEN), and the newly developed clustered regularly interspaced short palindromic repeats (CRISPR)/Cas system, have offered unprecedented possibilities of precise gene editing in a variety of organisms, which is promising for not only basic researches but also therapeutic studies of human diseases. Nowadays, scientists have attempted to apply gene editing approaches to fight against HIV/AIDS, sickle cell anemia, β-thalassaemia, Fanconi anemia (FA), laminopathies, and other genetic diseases (Li et al., 2011; Liu et al., 2011; Liu et al., 2012; Xu et al., 2012; Hu et al., 2014; Liu et al., 2014; Mandal et al., 2014; Suzuki et al., 2014; Xie et al., 2014; Ousterout et al., 2015). Besides successful applications in somatic cells and pluripotent stem cells, gene editing techniques have also been used in animal embryos to produce gene modified rodents, pigs, and monkeys (Wang et al., 2013; Yang et al., 2013; Hai et al., 2014; Niu et al., 2014) to create valuable research models for human genetic diseases.

Dysfunction of mitochondria, the energy-producing organelle of eukaryotic cells, may lead to mitochondrial diseases with severe symptoms in many organs, such as Leber hereditary optic neuropathy (LHON), mitochondrial myopathy, encephalopathy, lactic acidosis and stroke-like episodes (MELAS), myoclonic epilepsy and ragged-red fibres (MERRF), etc. Some mitochondrial diseases arise from disorders of nuclear genes which are involved in mitochondrial metabolism as well as in the maintenance of mitochondrial DNA (mtDNA). It has been found that a number of mitochondrial diseases are caused by mutations in mtDNA, a multi-copy, circular dsDNA molecule which encodes 13 essential polypeptides of the mitochondrial respiratory chain as well as the necessary RNA machinery (2 rRNAs and 22 tRNAs) for mitochondrial protein translation (Taylor and Turnbull, 2005; Xu et al., 2013). Since mtDNA is exclusively transmitted through maternal inheritance, a traditional approach of therapy is to transfer the nuclear genomic DNA to a enucleated donor oocyte or zygote with the normal mtDNA (Paull et al., 2013; Tachibana et al., 2013; Wang et al., 2014). This approach involves the mtDNA from a third individual thereby may trigger both ethical and technical conflicts. The most recent report by Reddy et al., for the first time, has prevented the germ-line transmission of mitochondrial disease by selectively eliminating the mutant mtDNA *in situ* in oocytes and one-cell embryos (Reddy et al., 2015). Using mitochondria targeted restriction endonucleases, the authors first tested their system by selectively eliminating the mtDNA haplotype in mouse oocytes and one-cell embryos. Cheerfully, the progenies from the modified embryos were verified to be free of the mtDNA haplotype which was supposed to be selectively cut and degraded. After the successful manipulation in mouse oocytes and embryos, the authors subsequently succeeded in specifically reducing the mutant mtDNAs responsible for LHOND and NARP (neurogenic muscle weakness, ataxia, and retinitis pigmentosa) by applying mitochondria-targeted TALENs in artificial mammalian oocytes, which were derived by fusion of patient cells with mouse oocytes. The highly efficient targeting mutant mtDNA in both animal model and human cells demonstrated in this study is cheerful. As being widely commented in the field, this report may fundamentally shape the future development of mitochondrial disease therapies. It is exciting to imagine that in the future by applying this technology in human, healthy babies will be able to born from patient oocytes where most mutant mtDNA are cleaned and the copy number of residual mutant mtDNA is reduced to below the threshold needed for a disease manifestation. Compared with other mitochondrial replacement therapies currently under development, this new technology no longer requires donor oocytes from an independent individual, and is a less complex procedure which would be less traumatic to the oocytes. Despite all potential advantages discussed, the authors also warned a risk that the embryos might fail to implant in uterus when mtDNA copy number in the “edited” embryos was below a specific threshold. Nevertheless, it was the first time that gene editing of mtDNAs in germ-line cells is achieved, which may encourage and promote the future studies towards new therapies for maternally inherited mitochondrial diseases (Fig. 1).

**Figure 1:**
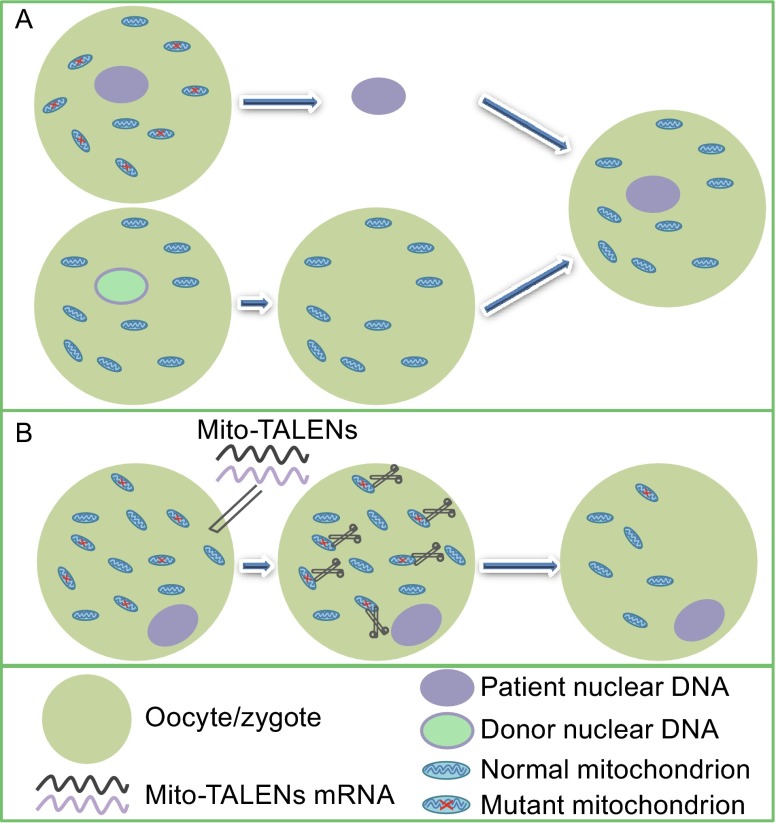
**A schematic representation of genetic approaches used for preventing mitochondrial DNA-based disease transmission in mammalian germ-line cells**. (A) The traditional mitochondrial replacement therapy is performed by transferring the patient nuclear DNA to the enucleated donor oocyte containing normal mtDNAs, or transferring the pronuclei from patient zygote to the enucleated healthy zygote of a third individual. (B) According to the newly developed approach, the mutant mtDNAs in the oocyte or zygote are selectively eliminated by mitochondrion-locating TALENs

Apart from the work of gene editing in mtDNA, a pioneering work editing nuclear DNA of human embryos has also been recently published and attracted tremendous attentions. Due to essential ethical and safety concerns, gene editing of nuclear DNA in human germ cells and embryos is traditionally discouraged or even banned in many countries. Liang et al. have reported for the first time testing the feasibility of CRISPR/Cas9 system in human tripronuclear zygotes (Liang et al., 2015), a type of embryo cells that would theoretically fail to develop *in vivo* and are routinely discarded in the conventional *in vitro* fertility (IVF) procedures. The authors applied the CRISPR/Cas9 system to edit the gene *HBB*, which encodes the human β-globin protein whose mutations are responsible for β-thalassaemia. Their results indicated that the CRISPR/Cas9-based gene editing was achievable in human tripronuclear zygotes. However, a notable amount of off-target effects of CRISPR/Cas9-based editing in human embryos was observed. Another major safety concern arose from the high rate of non-crossover homology directed repair (HDR) with adjacent endogenous *HBD* gene. The *HBD* gene whose sequence is highly homologous to the *HBB* gene might effectively compete with exogenous donor templates (or endogenous *HBB* wild-type sequence) for DNA recombination, resulting in unpredicted and unwanted mutations. More importantly, most edited embryos were genetically mosaic, which is both technically and ethically unacceptable for any clinical application. In view of the high efficiency of editing of *HBB* in the previous study (Xie et al., 2014), more efficient gRNAs for *HBB* gene than the ones tested in this study might exist, and the whole targeting strategy could be more cautiously optimized. Nevertheless, despite being controversial, this study sounds an alarm that CRISPR/Cas9-mediated precise gene editing technique is still premature and needs further investigation and improvement before any clinical application.

Although it has been evidenced that genome editing techniques have minimal impacts on genomic mutational load in human pluripotent stem cells using whole-genome sequencing (Smith et al., 2014; Suzuki et al., 2014; Veres et al., 2014; Yang et al., 2014), the safety issue of gene editing in germ line cells still deserves extreme cautions as such changes are permanent and heritable. For the mito-TALEN-based gene editing technique in germ cells or embryos, the concentration of injected mRNA should be precisely optimized in order to guarantee good incision efficiency while avoiding increasing off-target risks. On the other hand, in order to test if the mitochondria-locating TALEN could leak to nucleus which may lead to unpredicted incision and mutation on nuclear DNA, a thorough examination of whole-genome sequence is likely to be the ideal solution. Besides those mentioned challenges, we still don’t know whether this mito-TALEN-based gene editing technique would work efficiently on human oocytes or embryos containing mutant mtDNA. Therefore, even if ethical obstacles could be set aside, a comprehensive technical and safety evaluation is required before any clinic trial of this new technique.

Recently, mitochondrial DNA transfer was approved by UK government in February, 2015. This progress sets an excellent precedent for how to solve the controversy related to newly developed therapeutic technologies. Targeted gene editing of the mutant mitochondria in germ cells and embryos, which may eventually prevent the inheritance of devastating human genetic diseases, will definitely be of great interest and of benefit to the human society. Hence, though serious problems exist, it is important for both the public and scientific society to have an open mind, and keep the research in this field moving forward.
